# Transmission study of the Abdominal Compression plate (BodyFIX Diaphragm Control) for abdominal and stereotactic body radiotherapy

**DOI:** 10.1002/acm2.13373

**Published:** 2021-08-02

**Authors:** Hema Vaithianathan, Benjamin Harris

**Affiliations:** ^1^ Department of Radiation Oncology Olivia Newton John Cancer Research & Wellness Centre Austin Health Heidelberg Vic. Australia

**Keywords:** abdominal compression, dosimetry, motion management, stereotactic body radiotherapy (SBRT)

## Abstract

**Purpose:**

Abdominal Compression is one of the methods available to minimize breathing motion during stereotactic body radiotherapy (SBRT), particularly for abdominal malignancies. It might be necessary to treat some tumors with radiation entering through the compression device. One clinically available compression plate device (Elekta BodyFIX Diaphragm Control) was evaluated to understand its impact on dosimetry during clinical treatments.

**Methods:**

The BodyFIX compression device was CT scanned following departmental stereo scanning protocols. Treatment planning system (TPS) calculations were used to determine attenuation ratios through each section of the compression device: the outer frame, compression plate, and higher density couch fixation points and compression screw. TPS calculated skin doses where the compression plate will come in contact with the skin were recorded. All attenuation ratio fields were measured on an Elekta Versa HD linear accelerator. Where differences in attenuation were observed, TPS density overrides were found to bring calculated doses into agreement with measurement.

**Results:**

The compression plate and frame showed low dose attenuation (3%–4%). Only minor density overrides for the frame were required due to artefacts from the limited CT field‐of‐view. The high‐density materials in the couch fixation points resulted in higher attenuation (14%–20%). Similarly, the compression screw recorded very high attenuation (44%–65%), depending on the length of screw used. Skin doses assessed from the TPS calculations showed dose build‐up under the compression plate that would result in skin receiving the maximum dose.

**Conclusion:**

Compression devices can cause significant dose attenuation. Density overrides for TPS calculations are recommended for correcting attenuation in some sections of the device. High‐density structures like the fixation screw and frame fixation points create high levels of dosimetric uncertainty, and beam entry through those areas has been disallowed.

## 1 INTRODUCTION

During stereotactic body radiotherapy (SBRT) treatment, motion of internal organs as well as chest wall motion due to breathing are some of the sources of uncertainty in the dose delivered for lung and upper abdomen treatment areas such as the liver.^1^, ^2^ In particular, mitigating breathing motion allows for smaller target margins and dose escalation in the abdomen if motion in dose‐limiting normal tissues is also known and taken into account.^3^, ^4^

There are several common methods of limiting breathing motion, such as treating the patient in breath‐hold (voluntary or assisted) and/or treatment gating (only delivering radiation during part of the breathing cycle). Abdominal compression is commonly used to reduce the breathing motion where breath‐hold or gating is not suitable or unavailable.^5^, ^6^

Studies have shown the use of abdominal compression (such as plates or belts, etc.) can reduce the amplitude of abdominal tumor motion (liver and pancreas) by 36%–62% compared to free‐breathing, as measured using tumor surrogates on 4DCT or cone beam CT (CBCT).^3^, ^7^, ^8^, ^9^ While the impact of abdominal compression on normal tissue deformation is less clear compared to its impact on breathing motion, it has been shown the amount of deformation can be small (<5 mm) for most patients.^10^ In addition, motion mitigation studies have shown on 4D‐CBCT that abdominal compression can improve inter and intra‐fraction baseline reproducibility versus free breathing and that most amplitude changes in liver when using compression are small (80% less than 3 mm).^11^, ^12^

**FIGURE 1:**
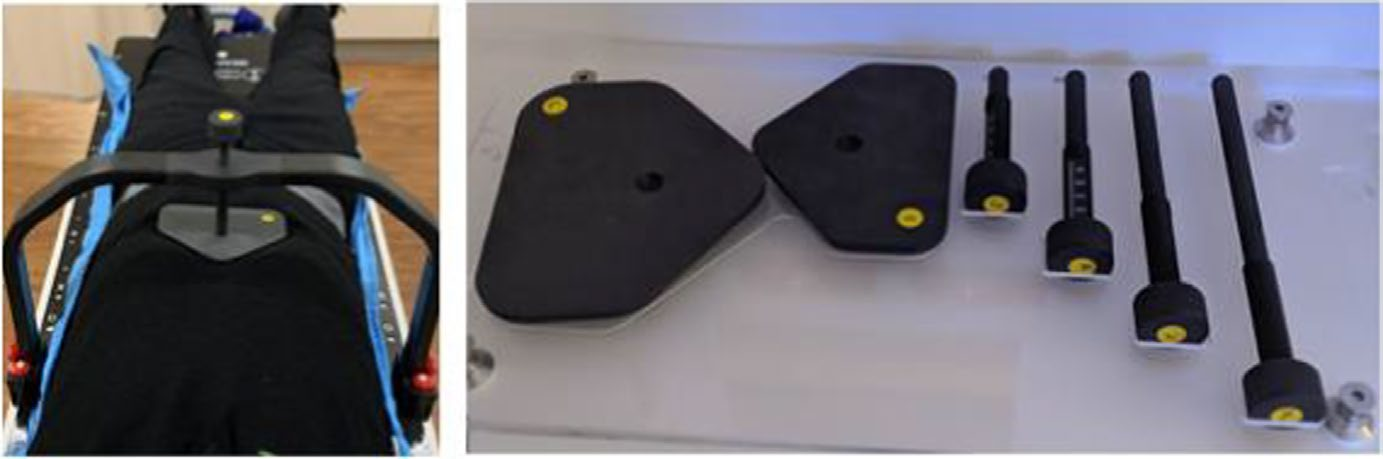
Accessories of the compression device: (left) frame, fixation screw, and compression plate in place for treatment; (right) two compression plate sizes and four fixation screw sizes to cover a wide variety of patient body sizes

The Elekta BodyFIX Diaphragm Control device is one of the abdominal compression devices that can be used during clinical treatments to minimize target motion due to diaphragm movement. The device (Figure 1) consists of a frame mountable on the treatment couch indexing points, a compression plate (two sizes: large, 17 × 14.2 × 1 cm, and small, 15 × 11.7 × 1 cm) placed on the patient's abdomen, and a fixation screw (four sizes: S, 11.6 cm length, M, 15.6 cm, L, 19.6 cm, and XL, 23.6 cm) to position the plate a set distance from the mounted frame.^13^ The various sizes are defined to cover a wide range of patient body sizes.^14^ The material for the screws and compression plates are defined as carbon fiber PA 6 GF 30 PVC by the vendor.^13^, ^14^

The fixation screw is driven down through the top of the mounted frame and then connects to the compression plate via a small indentation (to prevent sliding movement). As the screw is tightened, the plate is pressed down onto the patient's abdomen, with the plate shape designed to fit below the rib arc resulting in reducing breathing movements in the cranio‐caudal direction.

In this department, over the 18 months from January 2020 to July 2021, approximately 40% of stereo lung patients (19/50 total) were lower lobe, and half of those (10/50) were treated with breath hold gating techniques, with the remainder (9/50) treated free‐breathing. For stereo liver patients, breath hold gating techniques are predominantly used, with only 10% of stereo liver patients (4/37) treated free‐breathing. Following the implementation of the abdominal compression device, all patients that could not be treated with breath hold gating, and a small proportion of breath hold gating patients that were borderline ineligible (i.e. breath hold reproducibility close to tolerance or breath hold time length shorter than recommended), would be considered eligible for evaluation of abdominal compression device use. To date, for the first two months of use, three liver and lung patients have been treated or evaluated for treatment using abdominal compression.

During patient treatment, the treatment field geometry will be determined by the patient anatomy and target positions. While the majority of targets are expected to be located superiorly to the compression device, there is a distinct possibility that some may happen to fall beneath the compression device (particularly for liver lower lobe regions). In those cases, it may be necessary to allow fields to enter through the frame or other components to achieve adequate target dosimetry.

When any immobilization device could potentially be located within the treatment field area, the department requires relative dose attenuation measurements through the device and skin dose build‐up (if relevant) to be quantified by medical physicists. Dose calculation accuracy through the device is then verified in the treatment planning system (TPS), and if any variations are observed, electron density (ED) overrides applied in the TPS to more accurately represent the measured attenuation or skin dose. When available, these attenuation measurements and ED overrides as applicable are compared against vendor/device documentation, but in this case, the vendor‐provided attenuation through the device was defined as 1%,^13^ which did not define which region of the device this applied to. It is thus necessary to quantify the impact the compression device might have on treatment dosimetry and determine if dose calculations are sufficiently accurate for treatment.

## 2 METHODS

### 2.1 CT simulation

The frame, plate, and screw were scanned on a Siemens Somatom CT scanner (Definition AS). The departmental stereotactic abdominal protocol (120 kVp, 65 cm field‐of‐view (FOV), 1 mm slice thickness) was used for all scans. Solid water blocks of 30 × 30 × 24 cm were used as the measurement phantom. Two clinical combinations were scanned—XL screw with large plate and M screw with small plate. The XL screw represented the highest anticipated device attenuation, and the M screw was chosen to be typical for most patient treatments.

**FIGURE 2:**
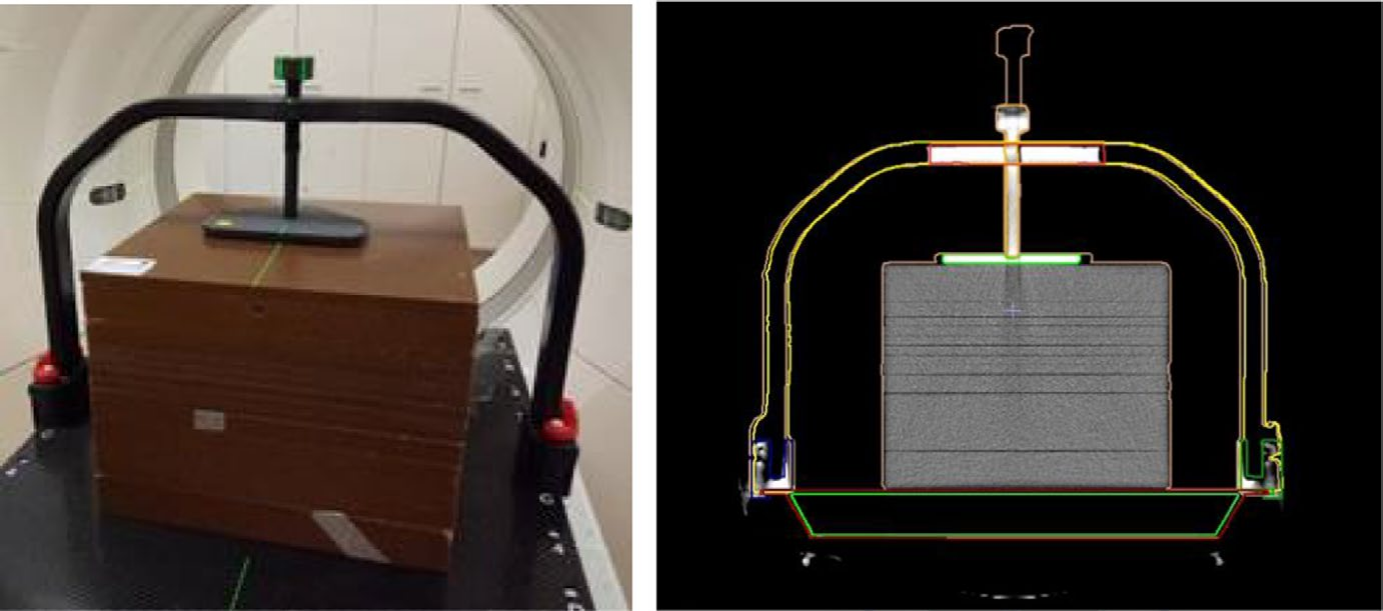
Phantom with the frame scanning in Siemens CT scanner and TPS Structures contoured in MIM and sent to Monaco for dosimetry

Due to the size of the frame and screw, the standard protocol's couch position resulted in part of the device being affected by scan artefacts at the edge of the CT scanner FOV. To determine the true shape of the frame and screw, multiple scans of the frame at different CT couch heights were acquired. The scans were then fused together, and the true shape's contours were defined in MIM Maestro (ver 6.9.5). Subsequently these contours were transferred onto the single standard planning CT dataset for all calculations. Figure 2 shows the setup in CT scanner as well the structures (screw, high density top, plate, frame and fixation parts.

### 2.2 TPS calculations

All calculations were performed using Elekta Monaco v5.11, with the clinical Monte Carlo algorithm (2 mm grid size, 0.3% standard deviation per control point to minimize statistical noise). The isocenter was placed at the lateral center of the water block phantom, in line with the compression screw center, and at a depth of 5 cm.

A series of 10 × 10 cm fields (100 MU per field, 6 and 10 MV photon beams, collimator and couch angles maintained at 0 degrees) was calculated for gantry angles that resulted in beam entry through each area of the compression device: through the frame, high‐density couch fixation, compression plate, high‐density top screw fixation and screw. Additionally, a subset of fields was recalculated with the isocenter shifted 4 and 3.2 cm superior to the compression screw center to check beam entry through the compression plate by itself or through the highest density parts of the couch fixation.

For all beams, two plans were calculated: one with the compression device in place, and one with the device contours overridden to air (electron density (ED) = 0.01). The dose to the isocenter was recorded for each calculation and were used to calculate the attenuation ratio through each part of the compression device.

### 2.3 Ion chamber measurements

**FIGURE 3:**
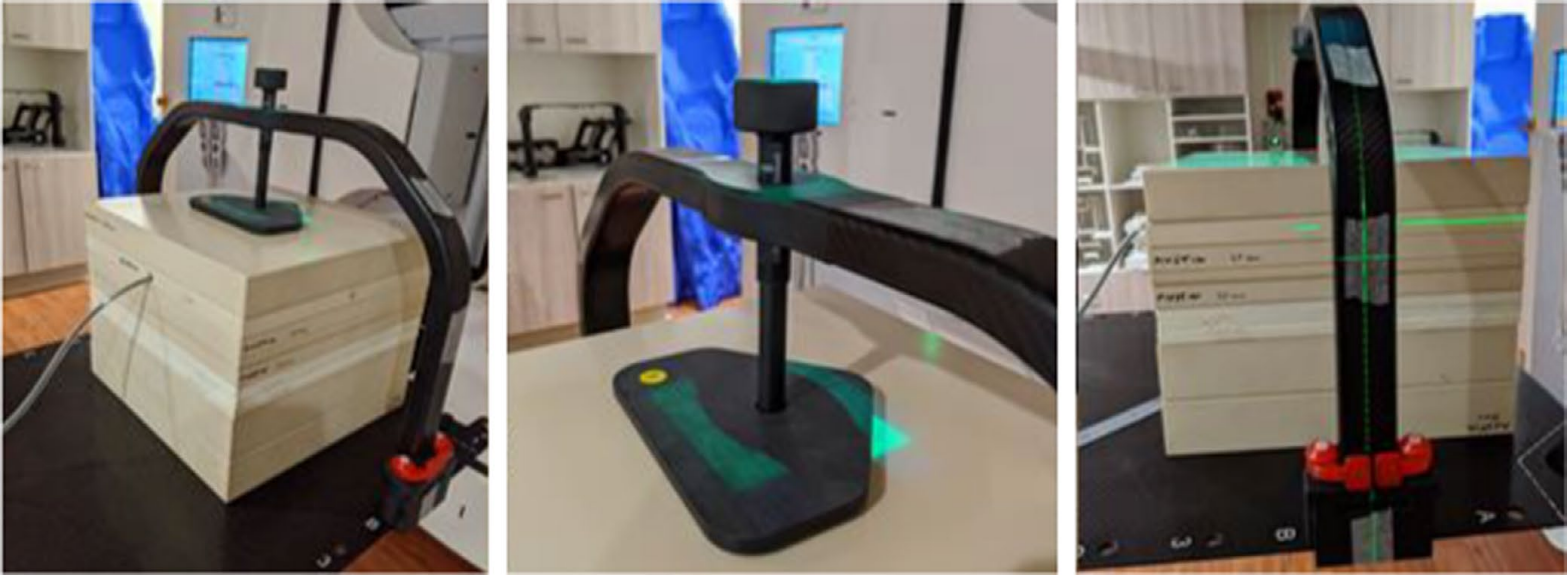
Phantom and device setup on the linac for measurement

All measurements were performed with a CC04 ion chamber and PTW Webline electrometer (−300V applied voltage) on two matched Elekta Versa HD linear accelerators. The CC04 chamber was positioned at linac isocenter, at 5 cm depth in Plastic Water phantom blocks (i.e. SSD 95 cm at gantry 0) and laterally centered to the middle of the treatment couch, matching TPS calculation conditions for both 6 and 10 MV photon beam measurements (Figure 3).

For gantry 0 measurement through the screw, 500 MUs were delivered to acquire enough ion chamber charge through the high‐density screw attenuation (and scaled back to 100 MU for comparison). For all other gantry angles matching the TPS calculations, 100 MU was delivered per beam. For the additional shifted isocenter readings, the couch and block phantom were shifted while leaving the compression device in its original position. Several readings were taken for each gantry angle measurement and over multiple repeat setups on both linear accelerators to minimize measurement and setup uncertainties.

The measurements without compression device were taken in the same sessions, using the same ion chamber and phantom block setup but with all parts of the compression device removed from the treatment area.

### 2.4 TPS density overrides and clinical plan validation

Following the ion chamber attenuation measurements, where differences between measured and calculated attenuation ratios were observed, the TPS calculations were repeated with ED overrides applied to the appropriate compression device contour.

Clinical plan evaluation was performed by taking seven clinical stereotactic body radiotherapy plans (a mixture of lung, liver and abdominal node targets) and calculating dose through the compression device contours and ED overridden structures. The dose delivered through the compression device was then measured using the same ion chamber setup as before and evaluated against the TPS calculated doses.

To check the ED override applicability to other energies, a subset of the fields used previously passing through the areas of highest attenuation (with the largest ED override corrections as required) were recalculated and remeasured using an independent 6MV‐FFF beam. TPS calculations were done using the same ED overrides as those determined using the initial 6 MV/10 MV fields.

Skin dose through the compression plate (where the compression device would come into contact with the patient) was evaluated in the TPS by comparing beam entry doses with and without the plate at the surface of the phantom.

## 3 RESULTS

**FIGURE 4:**
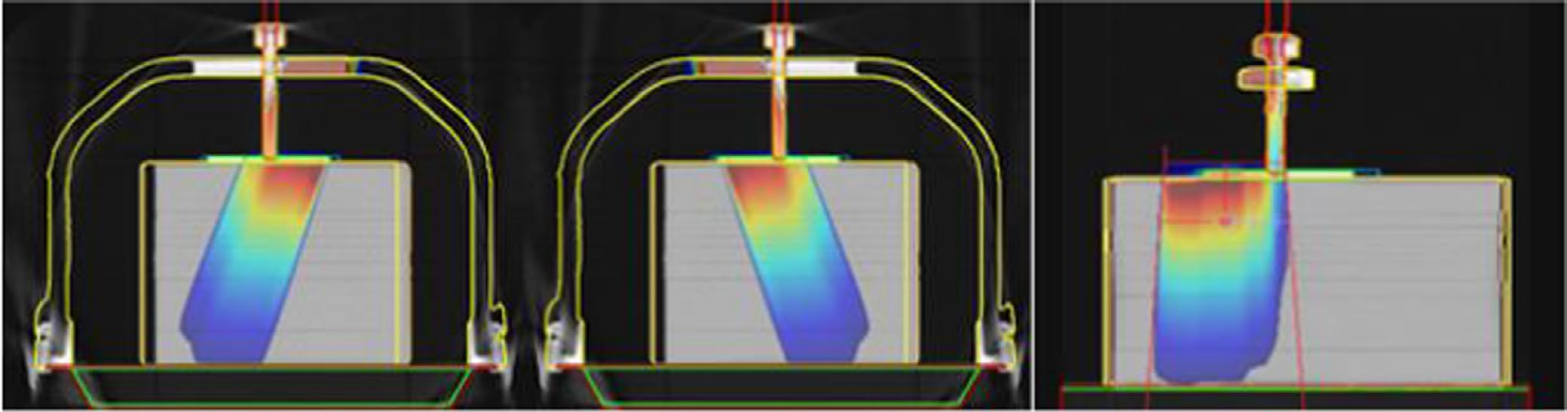
Beam arrangement through G20 (left) and G340 (centre), and (right) a sagittal view through the compression plate alone, offset 4 cm superior to the fixation screw centre

Table 1 shows the TPS calculated and measured transmission factors for the compression plate alone, with the ion chamber positioned 4 cm superior to the center of the frame and compression screw, as per Figure 4 (right).

**TABLE 1:** Transmission factors through the compression plate alone

No ED override	Energy	TPS transmission factor	measured transmission factor	% diff
G 0	6X	0.964	0.969	−0.5
	10X	0.976	0.979	−0.3

No ED override was required for the compression plate alone. Measurements taken through both the small and large compression plates were found to have the same transmission factor (within 1%) for these relative transmission measurements as calculated in the TPS.

TPS calculated and measured transmission factors through the compression plate and top high‐density screw support area, as shown in Figure 4 (left and center) are tabulated in Table 2. An ED override of 1.25 for the top screw support area (overriding the scanned value of ED 1.15 to 1.30) was found to be a reasonable match for measured transmission factors. The transmission ratios were found to be equivalent on either side of the device (within 1%).

**TABLE 2:** Transmission factors through the plate and screw support area

ED 1.25 override	TPS calculated transmission factor	Measured transmission factor	% diff
6X	0.844	0.850	−0.7
10X	0.871	0.875	−0.4

**FIGURE 5:**
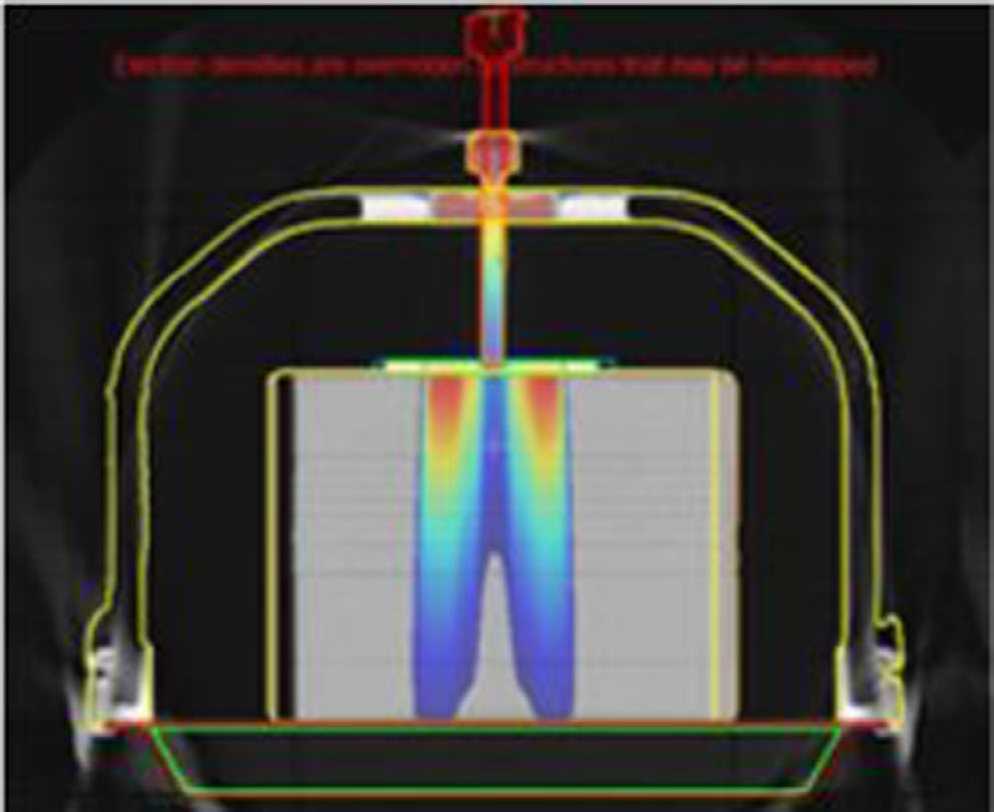
Beam arrangement through G0 at the fixation screw

In Table 3 the TPS calculated and measured transmission factors through the compression screw are as shown in Figure 5 . The 1.25 ED override for the screw support area was used for these TPS calculations. An ED override value of 1.20 for the compression screw (overriding the scanned value range of ED 0.8–1.2) was found to be agreeing with measured transmission factors (within 3%). Due to the high level of attenuation and complex setup for measurement, multiple repeat readings on different linear accelerators were used to limit the measurement setup uncertainty.

**TABLE 3:** Transmission factors through the compression screw

ED 1.20 override	TPS calculated transmission factor	Measured transmission factor	% diff
Medium screw 6X	0.481	0.475	1.2
Medium screw 10X	0.557	0.545	2.2
Long screw 6X	0.347	0.357	−2.9
Long screw 10X	0.425	0.430	−1.1

**FIGURE 6:**
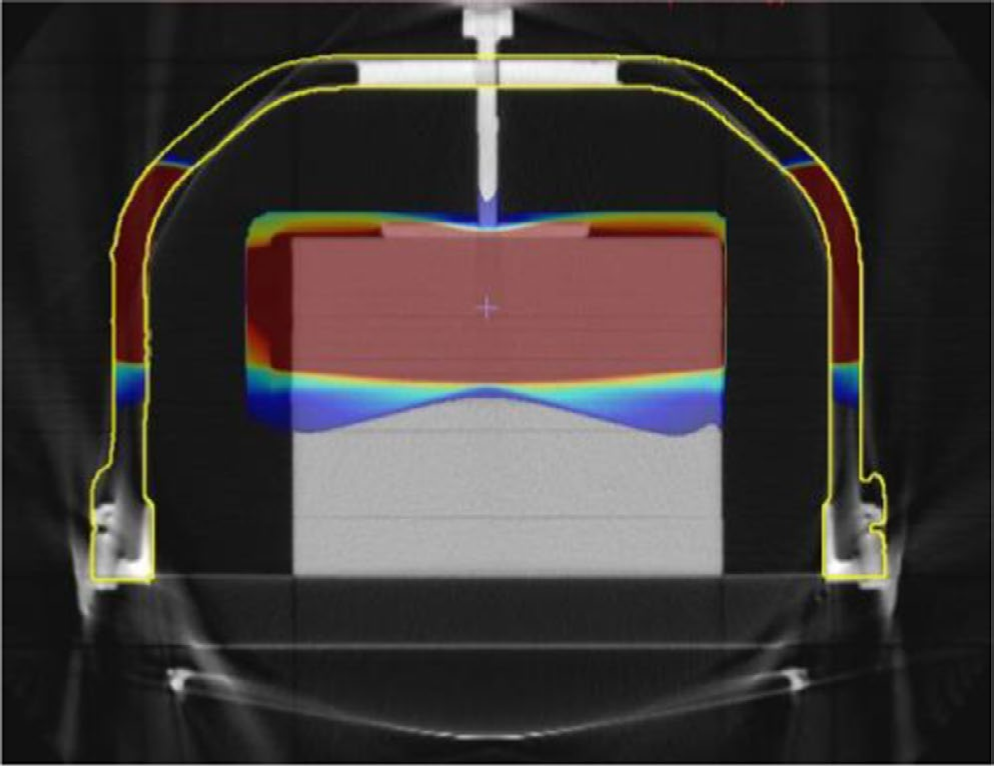
Beam arrangement through the frame alone (lateral entrance

Table 4 shows the TPS calculated and measured transmission factors through the low‐density outer frame only, as shown in Figure 6. Transmission factors were evaluated at G90/G270 and G75/G285, where initial test calculations had shown the greatest range in transmission factors through the frame. For this subset of evaluated gantry angles, an ED override of 0.30 was agreeing well with measured transmission ratios (within 1%). The greatest improvement was observed in areas where CT Field‐of‐View artefacts had obscured the true position and density of the frame.

**TABLE 4:** Transmission factors through the frame

ED 0.30 override	TPS calculated average (range) transmission factors	Measured average (range) transmission factors	% diff average (range)
6X	0.974 (0.969–0.980)	0.976 (0.974–0.979)	−0.2 (−0.3 to +0.5)
10X	0.978 (0.975–0.981)	0.980 (0.978–0.983)	−0.2 (−0.3 to +0.1)

**FIGURE 7:**
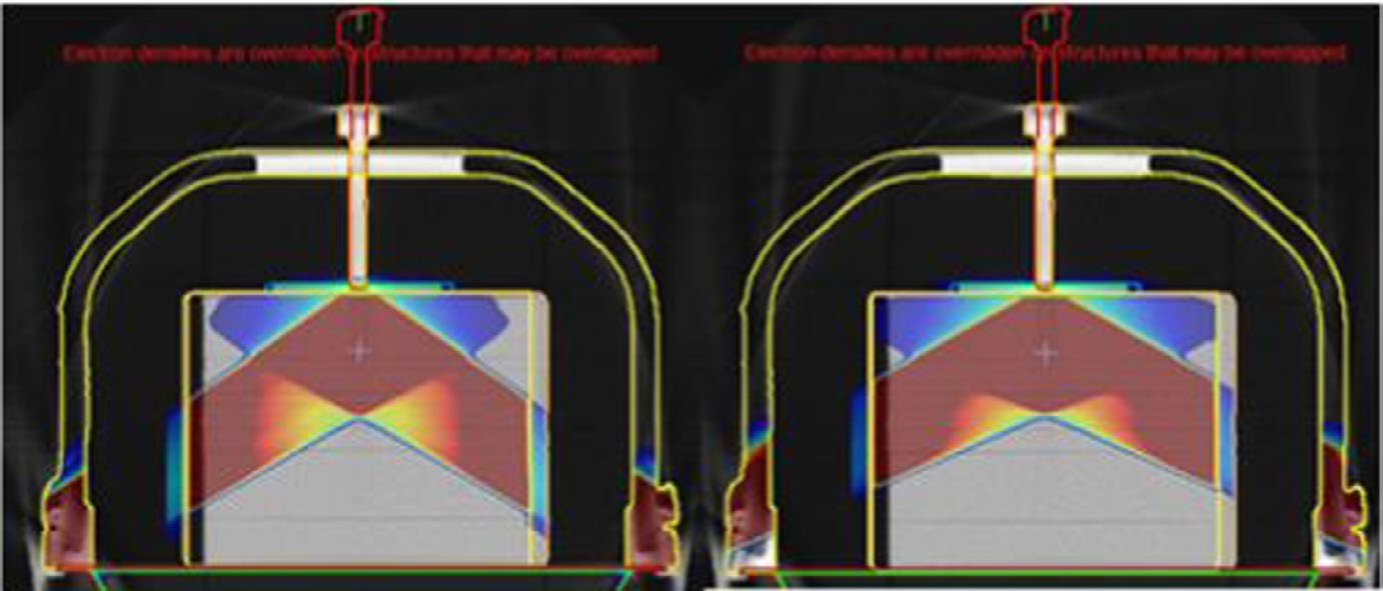
Beam arrangement through the couch fixation region for beams aligned with the centre of the fixation screw (left) and offset 3.2 cm superiorly (right) to cover the region’s high density variations

Table 5 has the TPS calculated and measured transmission factors through the high‐density couch fixation points. Transmission factors were evaluated at G125/235 (Figure 7) at isocenter (centered on the screw) and at 3.2 cm superior to the center of the screw, based on where the highest density structures were observed in the CT scan.

**TABLE 5:** Transmission factors through the couch fixation points

ED 1.00 override	TPS calculated transmission factor	Measured transmission factor	% diff
6X—G125, iso	0.833	0.829	0.5
10X—G125, iso	0.865	0.858	0.8
6X—G235, iso	0.819	0.833	−1.7
10X—G235, iso	0.853	0.861	−1.9
6X—G125, 3.2 cm superior	0.822	0.791	3.7
10X—G125, 3.2 cm superior	0.851	0.820	3.6
6X—G235, 3.2 cm superior	0.808	0.794	1.7
10X—G235, 3.2 cm superior	0.856	0.822	3.9

An ED override value of 1.00 for the couch fixation parts (overriding the scanned value range of ED 0.8–1.5) was found to be a reasonable match for measured transmission factors (difference between TPS calculated and measured transmission factors was within 2% at the isocenter slice, while the agreement was within 4% at 3.2 cm superior). Due to the high level of attenuation and complex setup for measurement (including multiple small high‐density objects inside the fixation point structures), repeat readings on different linear accelerators were used to again limit the measurement setup uncertainty.

Using the ED overrides calculated above, a subset of calculations with beam entering at gantry angles through the highest density areas (screw and couch fixation points) were done with the clinical 6MV‐FFF model and these plans were measured. The results of these measurements using 6FFF beams entering through the screw and the fixation parts of the frame are given in Table 6. The results with an independent 6MV‐FFF beam through the highest attenuation areas with large ED overrides showed consistent results with the 6X/10X results.

**TABLE 6:** Transmission factors for 6FFF calculations (all density overrides applied)

6FFF—subset of fields	TPS calculated transmission factor	Measured transmission factor	% diff
G0 M screw	0.488	0.478	2.3
G125	0.825	0.829	−0.4
G235	0.830	0.847	−2.0

Table 7 summarizes the seven clinical plans measured through the compression plate device, with all ED overrides from above applied. All evaluated plans agreed with the TPS calculated dose to within a 4% range. The measured points included both high target dose and low avoidance dose areas, depending on the plan's dose geometry.

**TABLE 7:** Measured doses for clinical stereotactic plans through the device

Patient and dose details	TPS calculated dose (Gy) at ISO	Measured dose (Gy) at ISO	% diff
VMAT 6FFF Liver 40 Gy/5#	9.15	8.81	3.7
VMAT 6x Lung 36 Gy/3#	5.69	5.88	1.0
VMAT 6x Liver 25 Gy/5#	5.66	5.74	−1.4
VMAT 6x Node SV L 40 Gy/5#	8.56	8.76	−2.3
DCAT 6x Lung 48 Gy/4#	11.99	11.81	1.5
VMAT 6x Node 40 Gy/5#	1.39	1.42	−1.7
VMAT 6x Node 40 Gy/5#	1.65	1.59	3.4

**FIGURE 8:**
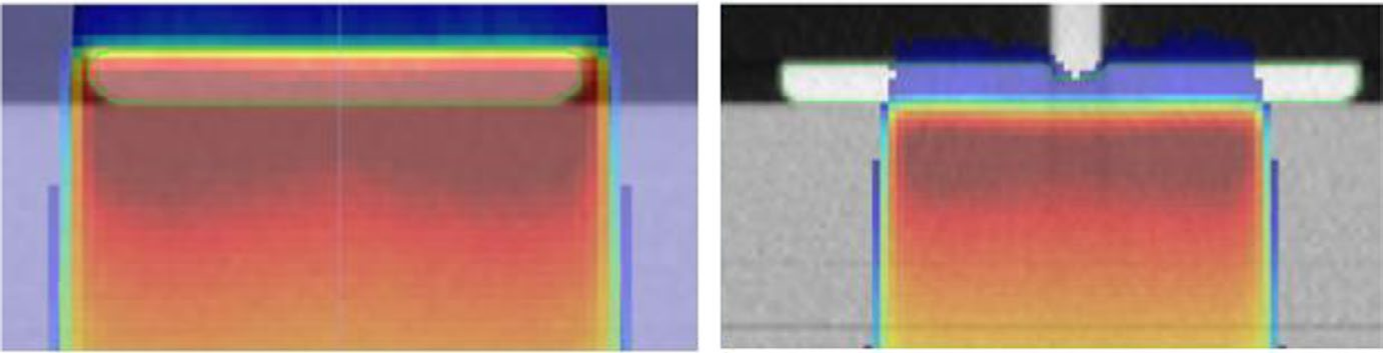
Demonstration of TPS skin dose calculations (6 MV photon beam), with the compression plate in place (left) and with the plate density overridden to air (right)

Figure 8 shows examples of skin dose at the phantom surface, with and without (i.e. plate density overridden to air) the compression plate in place. Since the compression plate is in contact with patient's skin, the TPS calculated skin dose is increased to approximately 99% of the maximum dose for 6x and 100% for 10x beams respectively from Gantry 0.

## 4 DISCUSSION

During clinical treatments, depending on the technique used, beams may enter through any part of the compression device. Hence, it is necessary to understand the effect of this frame in the path of the beam during clinical treatments. The measurements were done to assess the transmission of all sections of the device: the low‐density frame, the compression plate that would touch the patient's skin surface, and the high‐density support, screw and couch fixation points. Gantry angles were selected to cover the range of each of those sections on either side of the frame.

It was observed during CT simulation the large size of the frame and screw could result in FOV artefacts affecting the outer geometry (in particular, for the most distant screw and the couch fixation points, as well as the outer frame). This is particularly significant for the couch fixation parts as they were found to be highly heterogeneous, with multiple small high‐density subsections, so any artefacts will have significant effects on dose calculation accuracy.

The highest dose attenuation was found to be through the compression screws. Transmission at gantry 0 ranged from 35% to 48% for 6X, and 43% to 56% for 10X, for the M and XL length screws respectively. For these screw lengths, an ED override of 1.20 was found to be agreeing well with the measurements (within 3%). It can be noted from Table 3 that a small consistent difference was observed between the screws. Several sources of uncertainty could be attributed to this, including the uncertainty in contouring true screw size due to the previously described CT FOV artefacts, manufacturing differences between screws (with the scanned HU values ranging between 0.8 and 1.2, inclusive of FOV artefacts), and the reasonable expectation of uncertainties in the measured values due to set‐up and positioning of the ion chamber beneath a highly attenuating small structure. Taking these uncertainties into account, the final ED override of 1.20 was considered to be a reasonable compromise between the two evaluated screws.

The accuracy of dose comparisons between measurement and calculation through the screw are estimated to be more uncertain than those through the more homogenous compression plate or low‐density frame areas. The screw was both highly attenuating and a relatively small object in the field projection, so the dose gradient beneath the screw was extremely steep. Based on examples of acceptability criteria or potential deviations for external dose calculations, the screw attenuation measurements could be considered in a complex geometry (or 3D inhomogeneity phantom) position.^15^ For this work, an acceptability criterion of 5% agreement between measurement and calculation was chosen for evaluation. With repeat measurements on multiple linear accelerators used to limit measurement uncertainty, the final ED override of 1.20 was within this range.

The screw structure will be in a different position for each patient depending on patient's size and location. Hence, the screw structure needs to be contoured for each patient position. Considering the uncertainties described earlier—the variability in screw position, likelihood of CT FOV artefacts, and complex structure for setup (i.e small positioning shifts will result in significant transmission differences)—the dosimetric accuracy of planned dose through the screw may not be acceptable for treatment. The vendor also recommended to allow the beam to pass through the plate only and avoid the screw and high density structures.^13^, ^14^ Hence, it has been recommended to avoid any beam entry through the screw for patient planning (e.g. by using an avoidance constraint on this structure during optimization).

The M and XL screws were used during initial commissioning to represent the range most expected to be used for treatment. In case, if the two other lengths (S and L) are being used more frequently, another ED override value might need to be determined for them. However, since the recommendation to avoid beam entry through the screw would still apply, it was considered not essential to determine ED override values and hence not included here.

Transmission through the high‐density couch fixation region was around 83% (6X), and 86% (10X) for the isocentric plane (aligned with the center of the compression screw) and around 80% (both 6X and 10X) for the plane 3.2 cm superior to the screw center. TPS calculated transmission ratios agreed well in general with measurements. For beams entering through the couch fixation parts, an ED override of 1.00 was found to be reasonable and agreed well with measured transmission ratios. The couch fixation parts include many small highly attenuating structures and can be significantly affected by CT‐FOV artefacts. Similar to the screw attenuation measurements, these small highly attenuating structures caused steep dose gradients, and the size and strength of the gradients were dependent on the amount of CT FOV artefact encountered. As such, these comparisons were considered complex geometry, and were evaluated with an acceptability criterion of 5%, which was met with the ED override applied. It has been recommended to also avoid all beam entry through the couch fixation parts where possible (e.g. by using avoidance constraints on these structures during plan optimization).

Dose transmission ratios agreed well (within 1%) through the low‐density frame and through the compression plate alone, as well for beams passing through both the higher density top screw support and compression plate sections (within 5%). For these areas, it was considered that using the nominated ED override values for the frame and top screw support, and no override for the compression plate, would be of sufficient accuracy for a clinical treatment plan.

When the nominated structure ED overrides were applied for TPS calculations, both the 6FFF test measurements and the series of clinical stereotactic plans delivered through the compression device showed good agreement (within 5%). It is to be noted that these test calculations included beam transmission through the compression screw and couch fixation points, the areas identified to have high levels of complexity and thus the areas more likely to disagree between calculation and delivery. However, for the range of test plans and doses evaluated, the agreement between measured and calculated doses suggests that plans calculated with the compression device and applicable ED overrides in place are not expected to show a systematic dose offset to the delivered treatment dose. In future, plans will include the recommendations to avoid allowing beam entry through the highest complexity regions, so the overall calculation and delivery agreement is expected to be maintained to within an acceptable range for treatment.

To illustrate the effect of not accounting for the compression device during treatment, one of the clinical plan case studies (6X liver stereo plan, 25 Gy prescription dose, 210° partial arc) was recalculated with the compression device excluded from the TPS calculation volume. For a point 3cm superior to the frame center, where only part of the device (the compression plate alone) was excluded, the dose difference was as low as 3% (26.8 Gy unattenuated vs. 25.9 Gy with the device). However, in a worst‐case scenario, for a point in line with the frame center (with beams passing through the compression device frame, plate, couch fixation, and high‐density screw), a point dose difference was as high as 11% (31.8 Gy vs. 28.2 Gy). While this is only a homogenous phantom calculation, a similar difference of this magnitude in a patient could result in clinically significant treatment outcomes. Including the compression device would also have overall plan quality impacts—by implementing plan avoidance of the high‐density areas, beam entry through the largest dose attenuation areas would be minimized, reducing the overall attenuation impact due to the compression device.

For skin dose through the compression plate, where it would be in contact with patient skin, it is evident that the plate acts as build‐up for the skin surface and the skin dose increases to the maximum dose for a beam entering through the plate. Studies have shown this effect for similar objects.^16^ This must be kept in mind while using the compression plate device for clinical treatments.

Very limited studies are found in literature on abdominal compression devices dosimetry. There are no studies found using Elekta BodyFIX system in the literature. Hence, it was not possible to compare our results with literature data. However, vendor correspondence (email) indicated our results agree with factory measurements. There was only one article found in the literature on dosimetry aspects of an in‐house abdominal compression plate.^17^ The authors have mentioned that the dosimetry aspects of the in‐house compression device will be assessed and no results are published.

The ED override values are considered to be specific to this specific combination of CT scanner, TPS calculation software, compression device, and linac model. A CT with a larger bore, with fewer FOV artefacts, may mean some of these ED overrides may not be required. Similarly, for different combinations of TPS systems and linear accelerators, studies have to be done to evaluate ED overrides and assessed for accurate dosimetry.

## 5 CONCLUSION

Dosimetric transmission through the Elekta BodyFIX Diaphragm Control compression device has been investigated. Transmission was least through the compression screw (35% to 56%). The couch fixation sections also showed reduced transmission (79% to 86%) and can have high levels of calculation and measurement uncertainty due to the very small high‐density subsections. The other regions such as the compression plate and the lower density frame recorded higher transmission (96% to 97%) and measurements agreed with TPS calculations to within 3%. These areas were considered more reasonable to allow beam entry through if required for treatment. It is noted that skin dose through the compression plate would increase to the maximum beam entry dose due to the plate acting as build‐up on the skin surface, so this must be taken into account if required to deliver dose through in a clinical treatment.

## CONFLICT OF INTEREST

None.
